# Searching for the Impact of Participation in Health and Health Research: Challenges and Methods

**DOI:** 10.1155/2018/9427452

**Published:** 2018-05-13

**Authors:** Janet Harris, Tina Cook, Lisa Gibbs, John Oetzel, Jon Salsberg, Carolynne Shinn, Jane Springett, Nina Wallerstein, Michael Wright

**Affiliations:** ^1^School of Health & Related Research, University of Sheffield, Sheffield S14DA, UK; ^2^Department of Disability and Education, Liverpool Hope University, Hope Park, Liverpool L16 9JD, UK; ^3^Centre for Health Equity, University of Melbourne, Level 5, 207 Bouverie Street, Carlton, VIC 3053, Australia; ^4^Waikato Management School, University of Waikato, Private Bag 3105, Hamilton 3240, New Zealand; ^5^Graduate Entry Medical School, University of Limerick, Limerick V94 T9PX, Ireland; ^6^New Hampshire Division of Health and Human Services, 105 Pleasant Street, Concord, NH 03301, USA; ^7^School of Public Health, University of Alberta, 3-289 Edmonton Clinic Health Academy, 11405-87 Ave., Edmonton, AB, Canada T6G 2C9; ^8^College of Population Health, University of New Mexico, Albuquerque, NM 87131, USA; ^9^Institute for Social Health, Catholic University of Applied Sciences Berlin, Kopenicker Allee 39-57, 10318 Berlin, Germany

## Abstract

Internationally, the interest in involving patients and the public in designing and delivering health interventions and researching their effectiveness is increasing. Several systematic reviews of participation in health research have recently been completed, which note a number of challenges in documenting the impact of participation. Challenges include working across stakeholders with different understandings of participation and levels of experience in reviewing; comparing heterogeneous populations and contexts; configuring findings from often thin descriptions of participation in academic papers; and dealing with different definitions of impact. This paper aims to advance methods for systematically reviewing the impact of participation in health research, drawing on recent systematic review guidance. Practical examples for dealing with issues at each stage of a review are provided based on recent experience. Recommendations for improving primary research on participation in health are offered and key points to consider during the review are summarised.

## 1. Introduction

Participation in developing and delivering health interventions is increasing as more health care is shifted to communities. Aging populations and the rise in chronic and long term conditions in resource-constrained health systems are triggering a shift from reactive, acute care to health promotion and illness prevention, with the aim of reducing health care costs [1, 2]. There have been calls for direct involvement of citizens in the development, implementation, and evaluation of health equity in policy, program, and service structure changes. As a result, the impact of participation in relation to health and community development is now being researched in a number of ways ranging from action research to randomised trials of effectiveness.

Participating in designing, delivering, and evaluating health interventions can potentially produce more relevant and appropriate interventions for different groups and communities [3]. Benefits include opportunities to contribute to setting research priorities, develop more user-focused research objectives, improve trajectories to impact, and develop research skills, while benefits to the people receiving the intervention range from user-friendly information, more appropriate strategies for recruitment, and user interpretations of findings [4]. Despite the many claims of benefit, systematic reviews of effectiveness to date have encountered challenges in relating participation to health impact [5–10]. A review assessing effectiveness of interventions driven by coalitions of governmental and nongovernmental organisations, for example, found they may improve health and reduce inequities among racial and ethnic minorities [8]. The effectiveness review was unable, however, to “provide a definitive answer” on the added value of such coalitions. Similarly O'Mara-Eves et al. [7] found that relating participation to health outcomes was difficult because experimental, quasi-experimental, and process evaluations provided only partial descriptions of structure and process. Without information on organisational contexts, political environments, and prevailing priorities, it becomes impossible to identify what influences the process and outcomes of interventions and initiatives using participatory approaches. These issues are compounded when attempting to synthesize evidence across countries, given the marked differences regarding public health and health care systems and the histories, understanding and practices of participation in health interventions and health research.

This paper takes a critical view toward systematic reviews of effectiveness that use health outcomes as the sole vehicle for defining the impact of participation. Participation in the process of designing and delivering health interventions can be a key factor in effectiveness. It is a complex phenomenon, leading to a wide range of short-term, intermediate, and longer term changes in health and well-being. The many dimensions of participation include building relationships; interacting with social and organisational networks; accessing and communicating with service providers; dealing with changes in the physical, social, and political environment in which people live and the structures of the system providing services. Further, effects of participation can be conceptualised at different levels and over various periods of time. For example, factors impacting health can occur at the level of individuals, relationships, community, and society [11]. Impact is also relative, dependent upon the stage of project development, and may increase over time when relationships lead to increasing involvement, trust, and communication [3].

The challenges of conducting reviews that explore the roles and impact of participation [9–15] include the following:

- Assembling teams that include people with experience of participation.
- Variation in descriptions of participation in health:
  - Inadequate reports of how and why context, relationships, group dynamics, or partnership synergy can influence outcomes.
  - Thin description of the structures and cultural understandings of participation in the country in which the study is taking place.
- Documenting the extent to which participation is reported and recognized as a possible factor influencing implementation and effect.
- Managing issues of quality, comparability, and synthesis when there is heterogeneity in terms of
  - different definitions of impact
  - different views on the importance of proximal and intermediate outcomes
  - different views on reporting unexpected and emergent end products.

Given the above challenges, this paper presents strategies that can be used when conducting systematic reviews examining the impact of participation. Our strategies are based on practical experiences reviewing and developing methods for qualitative and mixed methods synthesis, and experiences conducting studies looking at the impact of participation. The aim of the paper is to advance methods for systematically reviewing the wide-ranging impact of participation in health research. The paper is aimed at reviewers who need suggestions for dealing with a range of issues while conducting this type of reviews.

## 2. Methods

Methods for reviewing the impact of participation can follow the standard systematic review stages which include defining the intervention and outcomes, setting inclusion criteria, developing a preliminary theory for how the intervention ought to work, and judging quality and relevance of studies, data extraction, and synthesis [16]. This article is structured to show how at each stage these methods need to be adapted in order to address the challenges of conceptualising participation, identifying papers that include information on impact, extracting the information, and synthesizing the findings.

### 2.1. Assembling a Review Team

While review teams are traditionally comprised of people with expertise in systematic review methods and the topic, reviews of participation impact also need people who are experienced in using participatory approaches in research and knowledgeable about conceptualisation of impact. This would include nonacademic social actors who are participating as coresearchers in guiding the participatory research projects. For example, people with lived experience of the health issue who have participated in designing, delivering, and receiving health interventions are key to integrating experiential knowledge with theories of what works [17]. The different perspectives help to identify important elements of intervention and context. The process of facilitating the group needs to take into account the fact that people from academic backgrounds may be challenged to work with people that have a nonresearch background [4]. While local people with little experience of research may devalue their own contributions, seeing them to be less useful than academic knowledge, in other situations local people who are on research teams may have grown to see their own value. Facilitating the process needs to focus on drawing out different perspectives and reinforcing that all types of knowledge and experience are equally valued [18]. Practically, a diverse review team will view different elements of participation in research articles and be able to use their experience to interpret these elements when space restrictions in journal articles limit how much participation can be discussed. The diversity of the review team also ensures that a limited definition of participation and impact is not used for the review.

### 2.2. Describing the Intervention and Outcomes

Describing the intervention, which is the first stage of a systematic review, requires authors to sift through diverse definitions of participation [8, 19–21]. For the purposes of this paper, we are defining participation as the extent to which a person or a group of people exert influence on health research, health structures, practices, services, or policies that have an effect on their health and well-being. It has been described alternatively as patient and public involvement and community engagement, with a range of influence possible, from minimal to being an equal partner in the research decision-making. Searches from existing reviews can be used as a starting point, using preliminary searches to refine the key terms and clarify the concepts (see, e.g., [6, 7, 22]). Three questions can be used as a frame for guiding conceptualisation of impact, which can focus on the process of implementation, appropriateness, and effectiveness (see Table 1):

- Implementation: how do participatory approaches contribute to the process of designing and delivering the intervention?
- Appropriateness: to what extent does the approach to participation fit with the cultural, ethical or equity context?
- Effectiveness: do participatory approaches work?

Existing frameworks can also be used to decide upon “cut off” level for participation. The classic Arnstein [23] ladder of participation defines a continuum, from citizen control through cooptation, which followed Cornwall's parallel continuum [24] of six levels (Box 1). Research participation may also be coopted, yet continua in this arena focus more on the different types or extent of contribution of community stakeholders to the research process [3]. This can range from community members being involved at the first stage of defining the problem to being actively involved at all stages of the research including data interpretation and dissemination of findings for community action and benefit [25, 26].

In many research traditions, authors do not use this terminology to describe or classify participation. The description of participation therefore needs to be anchored in descriptions of* whether* different people are engaged,* who* is included in development of the research, and* how *different people contribute to designing and conducting the research. These descriptions can be organised by how people participate at each stage of designing, delivering, and evaluating interventions [29], or the processes that affect participation [27, 30].

The description needs to acknowledge that different types of participation may occur at different stages of a project [31]. Community engagement, for example, can range from outreach, through consultation, to collaboration and shared leadership [3]. Participation may be initiated from the bottom up in communities where there is a large stock of social capital, or it may be induced by policymakers and implemented by bureaucracies [32]. The aim for participation can be utilitarian, being primarily “a* means* (to accomplish the aims of a project more efficiently, effectively or cheaply)”. Conversely, it can aim to promote empowerment, being used as an* end*, “where the community or group sets up a process to control its own development” [33, 34]. Using this lens, participation can be conceptualised if research or programmes are done “on” communities, “in” community settings, or “with” community partners [35]. These heterogeneous different approaches need to be identified in order to make decisions on whether to include different approaches.

Most studies have defined impact as improvement in individual health outcomes [7]. In projects using participatory approaches, however, impact can also be experienced at group, organisational and/or systems level. Further, impact can be experienced at any stage of the project (Box 2).

The period of time for impact to occur needs to be considered, as changes in partnership processes can over time lead to longer term transformation of systems [10, 35, 36]. Impact therefore needs to be considered as a continuum where different effects are achieved at various levels over different lengths of time. This is quite different from an effectiveness review, which usually defines impact as the achievement of health outcomes at the end of an intervention.

### 2.3. Formulating a Review Question

Questions can focus on impact within projects or beyond projects. Within projects, the relative contribution of participation at various stages can be the focus of the review, or the review question can ask whether participation “works” in terms of achieving the desired health outcomes. Examples of possible questions are presented in Table 1. The examples are informed by existing theory and evidence of implementing systematic reviews [37–39].

There is also a “beyond project” set of review questions, assessing impact in terms of broader and more far reaching changes, often occurring after the original study has been completed.  Table 2 presents possible review questions to assess whether impact has been reported on social, economic, environmental, and health benefits for individuals, groups, communities, and systems. During the project, it is likely that researchers and local participants will be the main beneficiaries, while policymakers and nonacademics are the main beneficiaries after the study is completed [40].

### 2.4. Setting Inclusion Criteria

For reviews of participation, we would suggest that the focus of the review needs to be clarified using a scoping review. Scoping reviews are a way of mapping the territory of participation for a particular health topic. They not only serve develop definitions for participation and impact, but also help in making decisions about the boundaries of the review [41]. Four important questions to ask when scoping the literature are as follows:

1. What is the context in which participation takes place?
2. What is the aim of the participation?
3. What is the length of time over which the impact of participation is assessed?
4. What is the range of impacts therefore which are possible based on these three questions (i.e., short-term through long term, and individual through system/policy/structural changes).

Boundaries for what to include in the review should be set by assessing whether the aims for participation are similar across studies and whether diversity of context is an issue.

### 2.5. Dealing with Issues of Diverse Contexts

Context is important because participation can be very different across different localities and countries. For example, the US population is largely immigrant (whether recent or generations before) and includes the African-American legacy of the slave trade and American Indian tribes. This has spawned a specific understanding of “community” as sociocultural or political identity, often geographic or based in ethnic/minority group, but also including other shared identities such as disability or LGBTQ communities. These identities have promulgated specific forms of community organising and activism, strongly influenced by the early labour and later civil rights movements [42]. In the United States, a particular form of partnership is the academic-community research partnership (often labelled community-based participatory research or community-engaged research) that forms around the development of health interventions and policy initiatives, and research on their effectiveness. These collaborative partnerships typically involve academic researchers working with a diversity of community-based organisations or NGOs, community members and grassroots associations, policy makers, service providers, and other public and private agencies through all stages of the research process [14, 35]. In Australia, participation of stakeholders in health research is promoted through specific grants administered by the Australian government major research funding bodies to ensure the relevance of the research and translation into policy and practice. The model is most consistent with community-based participatory research with academics typically forming partnerships with government, industry, community, and health organisations.

In other countries, social participation is embedded within structures, for example, in Germany where municipal health promotion is being integrated via government sectors, nongovernmental organisations (NGOs), and citizen action groups working together to set priorities and define strategies for addressing health inequities. These efforts are supported by coordinating centers for health promotion in each state which obtain funding and guidance through structures created by the new Law on Prevention. In Brazil, though participatory precepts were well articulated in the 1960s with writings and activism inspired by Freire [43], social participation was codified in the 1988 constitution and in further redemocratization policies after dictatorship, including community councils for clinics and social determinant initiatives [44].

In contrast, while action research approaches are found within health research in the UK, there is little tradition of CBPR. INVOLVE, an organisation funded by the National Institute of Health Research (NIHR), was originally established mainly to recruit more people into research studies. It now supports active public involvement, defining public involvement as “research being carried out* ‘with'* or* ‘by'* members of the public rather than* “to,” “about,”* or* “for”* them” [45]. In addition the UK has established Collaborations for Leadership in Applied Health Research and Care (CLAHRCs) their role being to bring together Universities, local health and social care organisations, the National Health Service, and citizens. If an international review is proposed, then these very different histories need to be taken into account as they may reflect different forms and understandings of participation.

In each of these contexts, the aims of participation may be similar or different. Tables 1 and 2 can be used to categorise aims for the various studies and make decisions about which aims to include in the review.

The period of time covered by the project is important because it is related to different types of impact. During the project, impact directly related to the research may be created by those who are on the research team. Other activities may also be triggered, causing an indirect ripple effect. “Beyond project” impact is created by nonacademic partners such as policy makers and community members who use the learning to inform decisions and programme development. It is rare to find short-term and longer term impact in one publication, unless a journal is devoted to reporting a single project [46, 47].

Journals often require authors to publish methodology and results for intervention studies separately. If included articles are limited to those that report only health outcomes, reviewers will be working with articles where “years of partnership development and collaboration must be distilled to few words in a small number of journals willing to publish this more descriptive science” [6]. Articles reporting on longer term impact, as well as those describing process, may not be indexed to the original study because they are seen as separate. This has implications for searching, as a straightforward search on outcomes will rarely produce citations for process or longer term participation impact.

**Figure 1:**
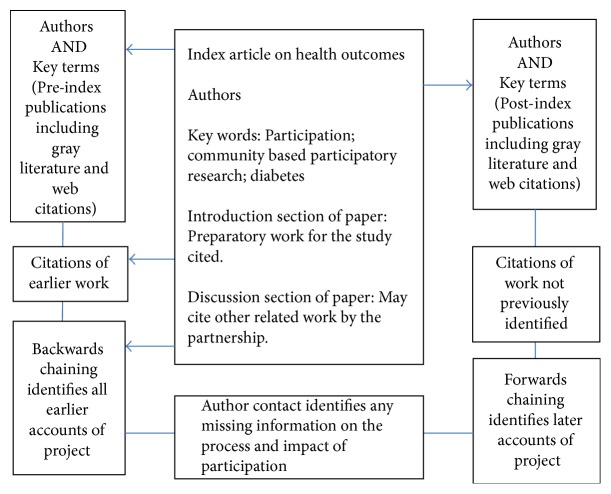
Cluster searching: a worked example.

A method called cluster searching can be used to identify all documents related to a particular project in order to trace pathways to impact [48]. Cluster searching is an iterative process. As shown in Figure 1, forwards and backwards chaining is done using the relevant article, the index paper, to identify all related materials. If it is possible to cluster papers on process, outcomes, and impact, a rich picture can be produced tracing the pathway from participation to impact [22].

Mapping what exists in terms of participation aims and contexts and periods of time to achieve various impacts will lead to being able to answer the question what is the range of impacts which is possible based on these three questions (i.e., short-term through long term, and individual through system/policy/structural changes).

The scoping review will produce information on the types of studies that have been published on participation, which can be used to decide upon the type of systematic review that can be conducted. As of 2009, 14 different systematic review types had been identified [49] and the number continues to rise. As studies exploring participation in health research are relatively new, it is likely that the most appropriate review types will involve (a) mapping, where an overview is presented and research gaps are identified; (b) qualitative reviewing where constructs and themes are identified illustrating the contribution of participation to health research; or (c) mixed methods reviews that combine learning from both process and outcomes studies to relate participation and intervention.

## 3. Developing a Preliminary Theory for Impact

As yet, there are no guiding conceptual frameworks for the relationship between participation and impact in health research. In both primary studies and systematic reviews, a preliminary conceptual map of how participation works can be developed using existing research and stakeholder experiences. A theoretical or conceptual framework can be developed that proposes general relationships between participation and impact (see, e.g., [50]). Alternatively, a logic model can be developed which illustrates the relationships between participation, research design, implementation, and outcomes for specific populations in a given context [51–53]. Reviewers can develop their own model or use or adapt a preexisting framework such as the CBPR conceptual model, which suggests that context influences participatory processes, which then influence the interventions and research undertaken, to ultimately contribute to a range of outcomes [27, 35].

For example, in our review of patient and wider community involvement in diabetes [54] we proposed that participation at different stages of the project could enhance the processes of clarifying problems related to diabetes, setting priorities for the research, designing the intervention, recruiting participants, collecting and analysing data, and disseminating learning (Table 3).

Frameworks and models can be used a priori to ensure that the search strategy explicitly looks for key concepts. They can also be used during the review to iteratively develop explanations for how participation works [55, 56].

## 4. Judging Quality and Relevance

When deciding which studies ought to be included in a review, concerns related to the quality of the primary research need to be addressed. Appraisal of quality generally asks whether the research was conducted in an ethical manner, whether it is relevant to practice or policy, the clarity of reporting, the coherence of the findings, and the appropriateness and rigour of the methods [57]. Filtering removes poor quality studies that may not enable decisions about the effectiveness of participatory research. Reviews that include studies with experimental or quasi-experimental research designs assessing the effectiveness of participation may use critical appraisal tools that are appropriate for the specific study design to assess methodological rigour.

Where the review question wants to know how and why something works, however, qualitative studies or studies with descriptive elements may be included on the grounds of relevance because they contribute to developing the explanation. In this instance, the appraisal process is used to make judgments about relevance. Studies containing “nuggets” of relevant explanation are included, rather than just including studies based on assessment of overall methodological quality [58].

## 5. Synthesizing Information on Participation

Participation adds to the complexity of an intervention, because it can mediate or moderate the effects of an intervention. Participation at one stage can create both positive and negative feedback loops, influencing the relative success of later stages. Where the components of an intervention are a poor fit with local contexts, participation can create a more receptive setting for the intervention. For all of these reasons, the approach to synthesis needs to be appropriate for complex interventions [59]. As noted in the section on review types, most of the research to date on participation is descriptive, mainly qualitative in nature with some studies on process and others focusing on outcomes. This type of research asks open-ended questions about participation. The approach is configurative aiming to generate theory and explore relationships. The recommended approaches to synthesis are outlined by Hannes [58] and include meta ethnography, thematic synthesis, critical interpretive synthesis, framework synthesis, realist synthesis, and narrative synthesis. The choice of approach is usually based on the material available and the skills of the review team. While it is beyond the scope of this article to explore synthesis in detail, there are several issues that will arise regardless of approach. These include selecting a framework for organising data, dealing with thin description of participation, establishing relationships between participation and outcomes, and mapping longer term impact.

At the synthesis stage, the original a priori logic model or theoretical framework can be used to organise data. Data extractors need to be trained in using an expanded and unconventional lens, as review authors have noted that information on the characteristics of partnerships and coalitions is often missing or inadequate, making it difficult to explain underlying mechanisms that promote health [9]. People may be motivated to participate when space is created for relationship building, where deliberation and dialogue is facilitated, and knowledge cocreation is promoted. Few primary studies, however, describe how the process fosters inclusivity and involvement. The important components of the project and descriptions of process may be scattered across documents. The challenge becomes one of configurations, where reviewers “read across” articles and report to piece together the story of how participation was promoted and how it contributed to impact. Further, where participation is an underlying storyline rather than the phenomenon being researched, reviewers will need to “read within” different sections of each paper. Data may be found in descriptions of the research problem, which are often outlined in the introduction section of a paper; accounts of getting people to participate; and reflections on the process found in Methods and Discussion. This process is referred to as “bricolage,” where the reviewers use the information “at-hand” to construct an explanation for participation that is based on information derived from different epistemologies [60].

Documenting the pathways to health impact/outcomes remains an important arena of inquiry, with challenges still in primary studies and reviews of participation and community engagement. For example, although O'Mara-Eves et al. [7] found solid evidence that community engagement was effective; their review was unable to explain why due to the lack of information in process studies. Further, they were challenged to explain the causal pathway “between community engagement, improvements in social capital/cohesion, and improvements in health outcomes (mortality, morbidity, and health behaviours).” [7, page 75].

We suggest that there are several reasons why causal pathways to health outcome are difficult to establish. First, health research interventions are usually conceived as consistently delivered, distinct and bounded activities that improve health outcomes for individuals independent of context. Recently there has been acknowledgement that the success of an intervention may depend on how well it is tailored to the individual [61] and the quality of the relationship between professional and client [22]. Where this “relational research” is missing, researchers are challenged to establish how social participation influences the trajectory of health interventions [62]. In community interventions, it is also difficult to draw boundaries around an intervention because they are events that both affect a wider community system and are affected by it [34, 63]. Instead of referring to interventions as linear pathways, we should be visualizing them as streams which are fed by events and relationships, which contribute in turn to larger changes in groups and networks. These collaborations have been described as the “steady process of mutual enlightenment born of longstanding exposure to each other's ideas” [22] but it is important to also search for instances where participation has led to unintended and potentially harmful consequences. What emerges may “have little or nothing to do with those targeted in the initial study” [6] and may include emergent outcomes which have very little to do with “health.” For example, reductions in crime and improvements in housing and social capital may occur at community and system levels [6, 15, 50, 64].

People who form partnerships and coalitions, combining their perspectives, knowledge, and skills can create a synergy where the whole becomes greater than the individual contributions [10]. This partnership synergy can contribute to intervention “blurring,” where interventions that were originally conceptualised as distinct and formal may over time become generic and embedded in informal networks of support, particularly when those that are delivering the intervention are part of the local community. This process raises the question of when to assess impact in terms of individual health outcomes, and the importance of tracking interventions over time.

Capturing the ripple effect at community and system-level is difficult and may require methods that go beyond traditional data extraction from journal articles. Explanations of impact can be obtained via participatory reviews, where stakeholders are involved throughout the review process in interpreting findings based on experience and knowledge [22]. Author interviews can be conducted, to explain relationships between participation, outcomes, and longer term impact that were not reported in primary studies [10]. Collaborative reflection across researchers on different projects has also recently been used to capture “between the lines” knowledge that may not be reflected in the main objectives or published studies [65].

These examples of the evolution of individual interventions, emergent individual outcomes, and synergy and ripple effects at community and systems level illustrate several key points about impact. First, impact is time-dependent in the sense that single studies usually report on a distinct point in the intervention. Whenever possible, this point needs to be documented to reduce the risk of synthesizing data that actually represents very different stages in the process. Second, impact occurs at all stages of a project, from its inception to completion, as well as leading to further spinoff projects [10]. When defining impact in a review, these key points need to be explicitly considered with statements about whether the review covers one or more levels, whether data will be synthesized for a specific stage of participation or as an evolutionary stream, and whether nonhealth outcomes are included.

A variety of issues need to be considered when determining the impact of a participatory intervention [35, 66, 67]. For example, historical issues of trust among the participating parties shape the nature of the participatory dynamics [27]. A poor fit between the proposed intervention and the context may lead to detrimental or ineffective processes and outcomes [68] and influence effectiveness of participatory interventions [66]. Readiness for change in organisations can influence the process of implementation [67]. One approach that integrates many of these issues and challenges is the CBPR conceptual model, https://cpr.unm.edu/research-projects/cpbr-project/cbpr-model.html  [35]. This model suggests paths from context to participation to intervention design to intermediate and distal outcomes. An empirical test of the model, using data from 200 CBPR US partnerships, illustrates two predominant paths: (a) where values/principles influences participation leading to synergy and then outcomes; and (b) where resources and shared control lead to increase community involvement in research leading to outcomes.

## 6. Recommendations

This article has introduced a range of methods that can be used or adapted to synthesize evidence on the impact of participation in health research. As methods continue to be developed, however, it is likely that this article will need to be updated within the next few years. For the present, we can conclude based on our experiences that researchers need to consider whether and how participation could affect the research. Ideally, this starts when framing the problem to be researched, but when that is not possible then participation issues need consideration at the design stage. More description is needed of how the participatory process contributes to the design of studies, their implementation, and the process of generating research knowledge. Primary research often contains inadequate descriptions of participation. Researchers could develop a priori logic models or theories that conceptualise how participation may affect the intervention and include evaluation of the process alongside findings. Authors could consider ways to provide more information about context, relationships, and participatory processes, by (a) integrating the information within the article, (b) publishing as a separate methods article, or (c) providing material supplementary to the publication.

We offer the following recommendations in the hope that systematic reviews of the impact of participation can address some of the limitations that have been encountered thus far.

Reviews of effectiveness should be open to recognizing that different forms of impact can be realized at various points in time during an intervention and that interventions can be greatly influenced by participation.

Reviews should consider the contextual issues (e.g., SES, salience of the health issue to the community, history of collaboration among stakeholders) and how these shape participation, relationships, research design, and intervention choices.

Reviews should consider the theoretical and conceptual mechanisms through which participation impacts interventions and outcomes, presenting these as a theoretical framework or logic model where possible.

Reviews should start with an explicit definition of participation. While this is good practice for all reviews, the definition needs to describe whether

- different levels and types of participation will be considered
- nonhealth outcomes will be included
- outcomes will include proximal, intermediate, and distal outcomes
- projects at different stages will be included
- how different cultural and system contexts will be dealt with
- how degree of alignment between the intervention and the context will be assessed
- the length and process of collaboration can be included as a criterion for selecting and extracting data from primary studies

When deciding what studies to include in the review, reviewers need to ask the following:

- Does the primary research actually meet the definition for engaged participation (conceptual security)?
- Is the amount of information on context, development of partnerships, and relationships between partnership processes and context adequately reported?
- Can strategies that compensate for thin description be used, such as
  - identifying all papers related to a specific project (cluster searching)?
  - contacting authors and/or conducting interviews with participants?
  - including participants in the process of systematic review (participatory review)?
- Should primary research that is of poor quality be included when it meets criteria for relevance, for example, including important information contributing to explanations of impact?

Reviewers need to ask whether differences between studies by cultural context, health or political system, partnership processes, type of population, intervention, or outcome warrant the analysis of impact by splitting papers into subgroups and conducting subgroup analysis. Further, when considering subgroup analysis decisions need to be made about whether to split papers by their participatory stance, with the assumption that projects with utilitarian aims may actually represent very different forms of participation than those that have aims of empowerment.

The use of a framework to categorise and judge the quality of participation could be considered, although none of the current frameworks have been used for a systematic review.

Last but not least, studies need to be viewed using a relational lens, examining processes of participation, how these processes foster relationships, and how relationships may lead to positive outcomes that reflect changes in community conditions, policies, and services, as well as those that are health.

## 7. Conclusions

Participation is an underlying but critical process which can affect health interventions. Methods are therefore needed to assess the potential impact of participation in health research beyond that of simply health outcomes. Reviews of the impact of participation are challenged by primary studies that focus on health outcomes with limited exploration of other outcomes and processes such as empowerment and capacity building. Social processes of participation that may have a major influence on impact of the interventions are rarely described in detail. Because the context and process of promoting participation are poorly reported, we recommend that review authors take a configurational approach to synthesizing evidence.

## Figures and Tables

**Box 1:**
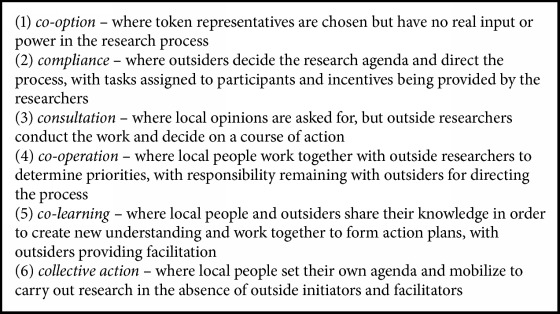
Levels of participation [24].

**Box 2:**
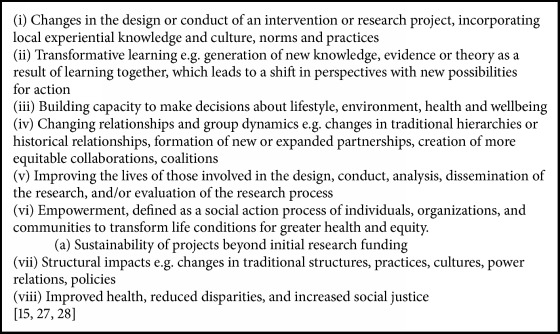
Different definitions of impact.

**Table 1:** Questions for reviews of the process of participation.

Type of inquiry	Types of review questions
Implementation inquiry: How do participatory approaches contribute to the process for designing and delivering the intervention or programme?	(i) How were people involved in deciding the components of the intervention? (ii) Were local people consulted or engaged in developing recruitment strategies? Did they do the recruiting? Were there barriers to recruitment that can be attributed to lack of engagement? (iii) Who participated? How many over time? Did the programme attract the target audience? (iv) What was the frequency, duration, and intensity of the intervention? Did it reflect the levels of participation that the target group would consider realistic or appropriate? (v) Did participants actually engage with the intervention? How did participants experience the intervention and did their experiences affect engagement? (vi) What were provider experiences of delivering the intervention? (vii) Was the intervention implemented as planned? Why or why not?

Appropriateness inquiry: To what extent does the approach to participation fit (or is it likely to fit) with the cultural, ethical or equity context?	(i) Is the approach appropriate, acceptable and accessible to people within their local context? (ii) How does the participatory intervention (potentially) impact on equity from both a positive and negative perspective for different population groups? (iii) Do the outcomes match the desired outcomes that are valued by the population? (iv) Are the desired outcomes consistent with people's priorities and/or beliefs? (v) What is the population's perception/experience of negative consequences of the intervention?

Effectiveness inquiry: Do participatory approaches work?	(i) What is the effectiveness of a community-based (intervention) compared to (interventions that do not use participatory approaches) for the population? (ii) Do the effects vary in relation to subgroups within the population? (iii) Do effects vary in relation to the country context and history of using participatory approaches in health?

Adapted from [37–39].

**Table 2:** Questions for reviews of the impact of participation.

Impact questions
(i) Did sharing of local experiential knowledge and culture, norms and practices instigate a change in the design or conduct of the intervention or research project? (ii) Did participation improve the lives of those involved in the design, conduct, analysis, evaluation and/or dissemination of the research? (iii) Did participation change historical relationships, group dynamics and traditional hierarchies or lead to more equitable partnerships and collaborations? (iv) Did participation lead to the formation of new or expanded partnerships, collaborations or coalitions? (v) Did participation create transformative learning, e.g., generation of new knowledge, evidence or theory, as a result of learning together? (vi) Did participation increase capacity on individual and/or collective levels to make decisions about lifestyle, environment, health and wellbeing? (vii) Did participation promote a social action process across individuals, organizations, and communities to transform life conditions for greater health and equity? (viii) Did participation have structural impacts, where changes occurred in traditional structures, practices, cultures, power relations, and policies? (ix) Did participation lead to improved health and wellbeing, reduced disparities, and increased social justice?

If there is adequate information, then the impact question would be included in the review and relevant data from papers would be used to answer it.

**Table 3:** An a priori theoretical framework for participation in diabetes research.

Propositions about involvement by stage of research
*Priority setting: *Getting people to identify the most important issues and participate in setting priorities for research will increase interest in participating in codesign of the intervention. Deciding priorities without involvement leads to questions on the relevance of the research.

*Proposal writing: *Involving people in writing proposals for funding increases collective ownership for research projects. Involving people after proposals are written risks less ownership and may make people feel that they are not equal partners in the project.

*Intervention design: *Asking people to help with the design of the intervention produces more culturally acceptable interventions, more appropriate approaches to recruitment, and more user-friendly information and tools. Excluding people from the design process may lead to project information that is difficult to understand, less cultural acceptance, and lower recruitment rates.

*Implementation:* Involving people in (a) recruitment produces high recruitment rates because they are able to help participants understand the relevance and benefits of the research. (b) delivering the intervention may increase trust and communication, and foster relationships which lead to high retention rates and good levels of active participation. (c) data collection and analysis may produce additional insight into how and why an intervention works (or does not work).

*Dissemination:* Involvement at any stage (a) promotes understanding of the aims and benefits of the research, creates local ownership and likelihood that a local network is created to share what is learned, and (b) helps to ensure that findings is relevant and understandable.
